# A five-minute drainage assessment prevents reexploration for bleeding

**DOI:** 10.1016/j.xjon.2024.08.008

**Published:** 2024-08-27

**Authors:** Go Yamashita, Shingo Hirao, Atsushi Sugaya, Jiro Sakai, Tatsuhiko Komiya

**Affiliations:** Department of Cardiovascular Surgery, Kurashiki Central Hospital, Kurashiki, Japan

**Keywords:** reexploration, five-minute drainage assessment, cardiovascular surgery, tamponade, bleeding

## Abstract

**Objective:**

To evaluate the effectiveness of the five-minute drainage assessment (FMDA) in preventing reexploration for bleeding following cardiovascular surgery.

**Methods:**

This retrospective review included 1280 patients who underwent cardiovascular surgery between January 2017 and August 2021. Patients were divided into control (n = 695) and FMDA (n = 585) groups. The FMDA involved estimating the bleeding volume from 1 drainage tube every 5 minutes during sternal closure. Reexploration rates, postoperative bleeding volumes, and clinical outcomes were compared between the 2 groups.

**Results:**

The FMDA group had a significantly lower rate of reexploration for bleeding than the control group (2.2% vs 4.3%; *P* = .038). The median postoperative bleeding volume within 24 hours was significantly lower in the FMDA group compared to controls (630 mL vs 695 mL; *P* = .009). Multivariable logistic regression analysis demonstrated that the FMDA was independently associated with a reduced risk of reexploration for bleeding (odds ratio, 0.49; 95% confidence interval, 0.25-0.96; *P* = .037). The FMDA demonstrated good discriminatory ability for identifying patients at risk of reexploration (area under the receiver operating characteristic curve = 0.782), with an optimal cutoff of 21.0 mL.

**Conclusions:**

Implementation of the FMDA was associated with a significantly lower rate of reexploration for bleeding compared to the control group. The FMDA provides a simple and reproducible approach that can be readily adopted in surgical practice.

## Graphical abstract

**Figure:**
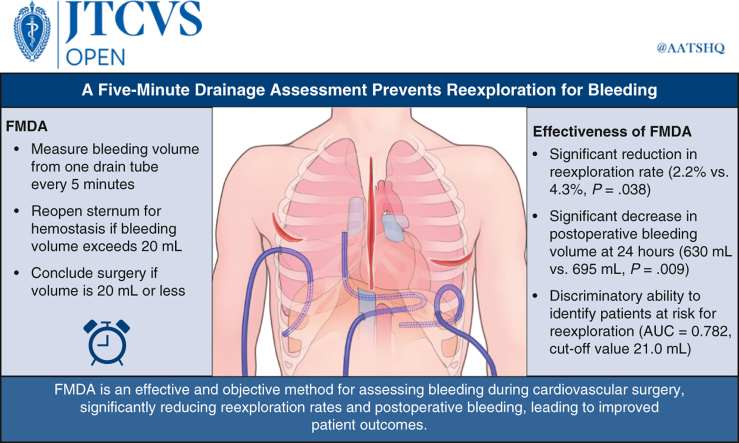

**Figure:**
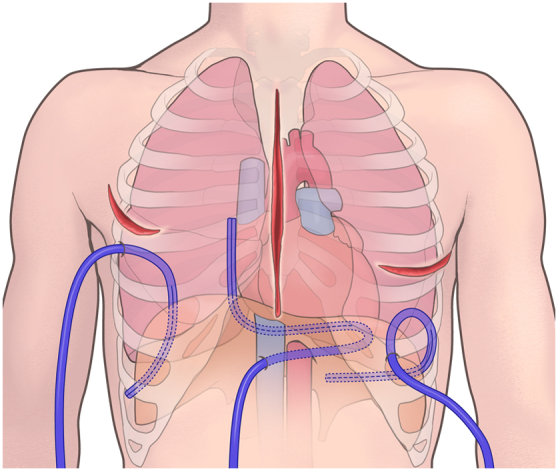
The five-minute drainage assessment avoids unnecessary reexploration for bleeding.

**Central Message**

The five-minute drainage assessment is a simple, objective method that effectively reduces the rate of reexploration for bleeding and the postoperative bleeding volume, leading to improved outcomes.

**Perspective**

The optimal criteria for determining the end of surgery applicable to all surgical approaches are unknown. The five-minute drainage assessment is a simple and effective method that fills this evidence gap, reducing reexploration rates and postoperative bleeding.

Postoperative bleeding is a significant complication following cardiovascular surgery and often necessitates reexploration, leading to increased morbidity and mortality.^1^^,^^2^ Reexploration for bleeding is associated with various adverse outcomes, including prolonged hospital stay^3^ and increased healthcare costs.^4^ Patients who undergo reexploration have a greater risk of postoperative complications, such as atrial fibrillation,^5^ mediastinitis,^6^ and deep sternal wound infection. Furthermore, these patients often require more significant transfusion volumes,^7^ which can lead to transfusion-related complications and poorer overall outcomes.^8^

Accurate assessment of bleeding during surgery is crucial for preventing postoperative hemorrhage and minimizing the need for reexploration.^9^ Traditional methods of assessing bleeding, such as visual estimation, are subjective and unreliable. Kunioka and colleagues^10^ reported a method for determining whether to close the chest by packing gauze into the pericardial cavity and measuring the weight of the gauze after 5 minutes. However, this method is applicable only to median sternotomy and cannot be applied to minimally invasive cardiac surgery (MICS), which has been increasingly used in recent years.^11^

In MICS, it is difficult to place gauze in the pericardial cavity, making it impossible to apply this method directly. Therefore, there is a need for an objective, standardized approach to assess bleeding during cardiovascular surgery that is adaptable to all procedures. We developed the five-minute drainage assessment (FMDA) using a single drainage tube to determine whether the surgery should be concluded. This study aimed to evaluate the effectiveness of the FMDA in preventing reexploration for bleeding following cardiovascular surgery.

## Materials and Methods

### Ethical Statement

This study adhered to the Declaration of Helsinki and its subsequent amendments. This retrospective observational study was conducted at a single center and was approved by the Institutional Review Board of Kurashiki Central Hospital (approval 4365; April 16, 2024). The requirement for informed consent was waived because of the retrospective nature of the study.

### Study Design and Patient Selection

**Figure 1:**
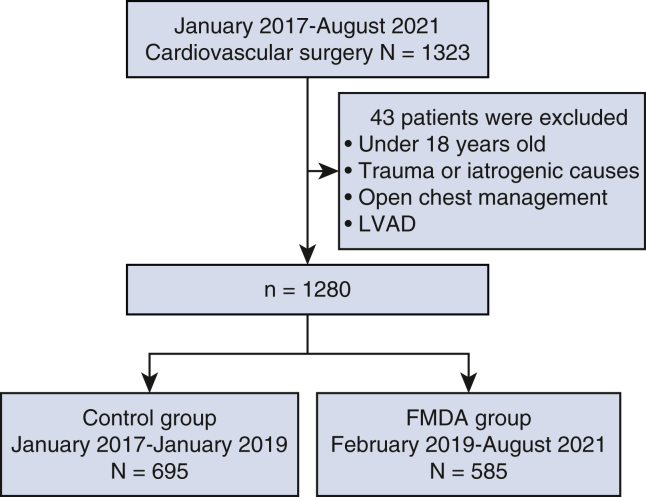
Patient selection and study flow. *LVAD*, Left ventricular assist device; *FMDA*, five-minute drainage assessment.

We enrolled 1323 patients who underwent cardiovascular surgery from Kurashiki Central Hospital between January 2017 and August 2021. Exclusion criteria were age <18 years, trauma/iatrogenic surgery, open chest surgery, and implanted left ventricular assistive device. After excluding 43 patients, 1280 patients were analyzed. Patients were divided into the control group (n = 695; January 2017 to January 2019) and the FMDA group (n = 585; February 2019 to August 2021) (Figure 1). We collected patient characteristics, operative details, and in-hospital complications from the electronic health record.

### FMDA Procedures and Criteria for Completing an Operation

**Figure 2:**
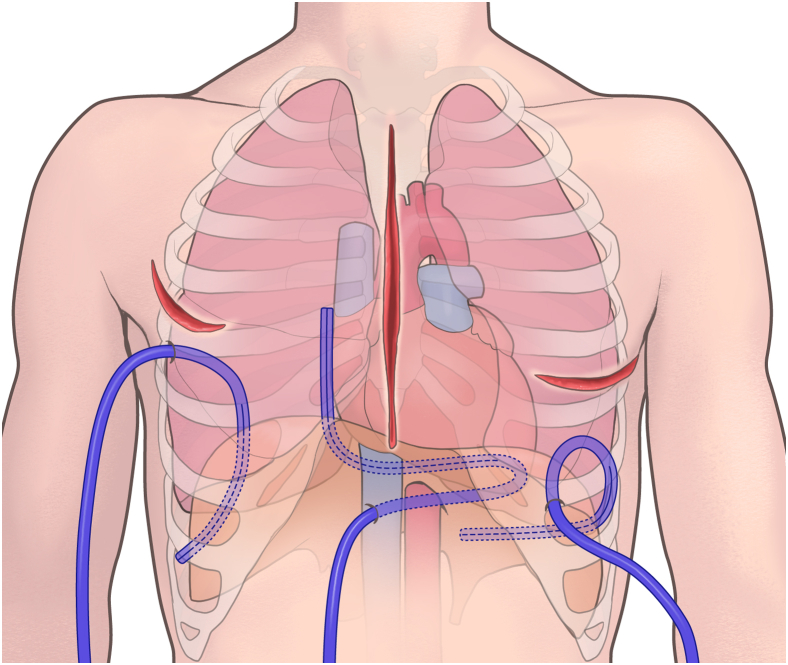
The drain location for the five-minute drainage assessment procedure depends on the surgical approach. A mediastinal drain for median sternotomy runs along the right side of the heart. A right pleural drain for right thoracotomy is positioned dorsally within the right chest cavity, and a left pleural drain for left thoracotomy is placed along the diaphragmatic surface of the left lung.

Introduced in 2019, the FMDA estimates bleeding from 1 drain every 5 minutes using the Sorin Xtra Autotransfusion System (LivaNova), starting from the moment the surgeon determined that the chest could be closed and began the sternal closure. The choice of a 19 Fr or 24 Fr Blake silicone drain for the FMDA procedure depended on the surgical approach. In the median sternotomy approach, the pericardial drain, which is placed along the right side of the right atrium, running from the apex of the heart, across the diaphragm surface, and toward the right atrium, is used for bleeding estimation. In the right thoracotomy approach, represented by MICS, the drain with its tip placed on the diaphragmatic surface, running along the dorsal side of the thoracic cavity, is used for the assessment. In left thoracotomy approaches, such as thoracoabdominal aortic replacement, a drain placed along the pulmonary diaphragmatic surface from the lateral thoracic cavity is used for evaluation (Figure 2).

If the bleeding volume measured by the FMDA exceeds 20 mL, the sternum is reopened to ensure hemostasis. In contrast, if the volume is ≤20 mL, the operation is concluded, and the patient is transferred to the intensive care unit. In cases of left or right thoracotomy, the FMDA procedure is initiated once closure of the intercostal incision has begun. Regardless of the approach, the FMDA is performed at least 4 or 5 times. Even if the initial FMDA volume is high, if the final measurement just before the end of the surgery is ≤20 mL, the surgery is concluded. Additionally, at the surgeon’s discretion, if the color of the drainage is pale, suggesting that the measured fluid consists primarily of irrigation water, closure may be performed even if the volume exceeds 20 mL (Video 1). More than 10 surgeons were involved in both the control and FMDA groups, reflecting our institution's standard practice.

### Postoperative Management and the Criteria for Reexploration After Cardiovascular Surgery

The decision to reexplore for bleeding is made by the surgeon based on our institution's criteria, which are derived from published studies.^12^, ^13^, ^14^ The following indications for reexploration due to bleeding were applied: chest tube output of 400 mL/hour, chest tube output of 200 mL/hour for 4 consecutive hours, significant hemodynamic compromise possibly related to bleeding, imaging evidence of a large clot, a large pericardial collection, and hemodynamic collapse. Nevertheless, the decision to reexplore is influenced by the surgeon’s own experience and preferences, patient characteristics, and knowledge of intraoperative factors, all of which result in individualized decision making that often may deviate from the formal criteria.

### Statistical Analysis

Statistical analyses were performed using EZR (Saitama Medical Center).^15^ Continuous variables are presented as mean ± SD or as median (interquartile range [IQR]) and compared using the Student *t* test or Mann-Whitney *U* test. Categorical variables are presented as count and percentage and compared using the χ^2^ or Fisher exact test. Normality was assessed using the Kolmogorov-Smirnov test. Non-normally distributed data were log-transformed. Pearson correlation coefficients were calculated for log-transformed drainage volume and the FMDA. The transformed data were then used to assess the statistical significance of the correlations between the variables. Multivariable logistic regression was used to analyze risk factors for reexploration. Receiver operating characteristic (ROC) curve analysis was performed to assess the predictive ability of the FMDA for identifying patients at risk of reexploration for bleeding or cardiac tamponade within 48 hours after surgery. The area under the ROC curve (AUC) was calculated to evaluate the overall discriminatory power of the FMDA. The optimal cutoff value for the FMDA was determined by maximizing the Youden index. A value of *P* < .05 was considered significant.

## Results

### Preoperative Patient Characteristics

The patient characteristics are shown in Table 1. There were no significant differences between the 2 groups in terms of demographics, comorbidities, or the use of pacemakers.

**Table 1:** Patient characteristics

Characteristic	Overall (N = 1280)	Control (N = 695)	FMDA (N = 585)	*P* value
Age, y, mean ± SD	68.1 ± 13.4	67.9 ± 13.1	68.4 ± 13.7	.542
Male sex, n (%)	813 (63.5)	441 (63.5)	372 (63.6)	>.99
Height, cm, mean ± SD	161.1 ± 9.7	161.1 ± 9.7	161.1 ± 9.8	.948
Weight, kg, mean ± SD	61.4 ± 13.1	61.5 ± 13.4	61.4 ± 12.8	.914
BSA, m^2^, median (IQR)	1.66 (1.51-1.79)	1.65 (1.51-1.80)	1.66 (1.51-1.79)	.965
Hypertension, n (%)	972 (75.9)	533 (76.7)	439 (73.5)	.512
Hyperlipidemia, n (%)	583 (45.5)	315 (45.3)	268 (45.8)	.866
Smoking, n (%)	645 (50.4)	350 (50.4)	295 (50.4)	.870
Diabetes, n (%)	306 (23.9)	167 (24.0)	139 (23.8)	.948
eGFR, mL/min/1.73 m^2^, mean ± SD	60.3 ± 36.9	61.7 ± 44.0	58.6 ± 26.2	.129
CKD (eGFR <45), n (%)	260 (20.3)	133 (19.1)	127 (21.7)	.265
Dialysis, n (%)	63 (4.9)	26 (3.7)	37 (6.3)	.040
Previous stroke, n (%)	145 (11.3)	87 (12.5)	58 (9.9)	.157
Atrial fibrillation, n (%)	174 (13.6)	99 (14.2)	75 (12.8)	.513
Pacemaker, n (%)	34 (2.7)	17 (2.4)	17 (2.9)	.728
PAD, n (%)	93 (7.3)	45 (6.5)	48 (8.2)	.237
COPD, n (%)	53 (4.1)	19 (2.7)	34 (5.8)	.006
Hemoglobin, g/dL, mean ± SD	12.8 ± 2.1	12.8 ± 2.1	12.8 ± 2.2	.684
BNP, mean ± SD	279.0 ± 604.0	306.4 ± 644.2	245.5 ± 549.7	.080
Albumin, g/dL, mean ± SD	3.77 ± 0.59	3.81 ± 0.58	3.73 ± 0.59	.013
Ejection fraction, %, mean ± SD	57.4 ± 11.5	57.3 ± 11.8	57.6 ± 11.2	.621
Antiplatelet therapy, n (%)	1 (0.6)	1 (1.0)	0	.426
Warfarin, n (%)	7 (4.4)	5 (5.2)	2 (3.3)	.577
DOAC, n (%)	10 (6.3)	9 (9.3)	1 (1.6)	.090

*FMDA*, Five-minute drainage assessment; *BSA*, body surface area; *IQR*, interquartile range; *eGFR*, estimated glomerular filtration rate; *CKD*, chronic kidney disease; *PAD*, peripheral artery disease; *COPD*, chronic obstructive pulmonary disease; *BNP*, brain natriuretic peptide; *DOAC*, direct oral anticoagulant.

### Perioperative Outcomes

**Figure E1:**
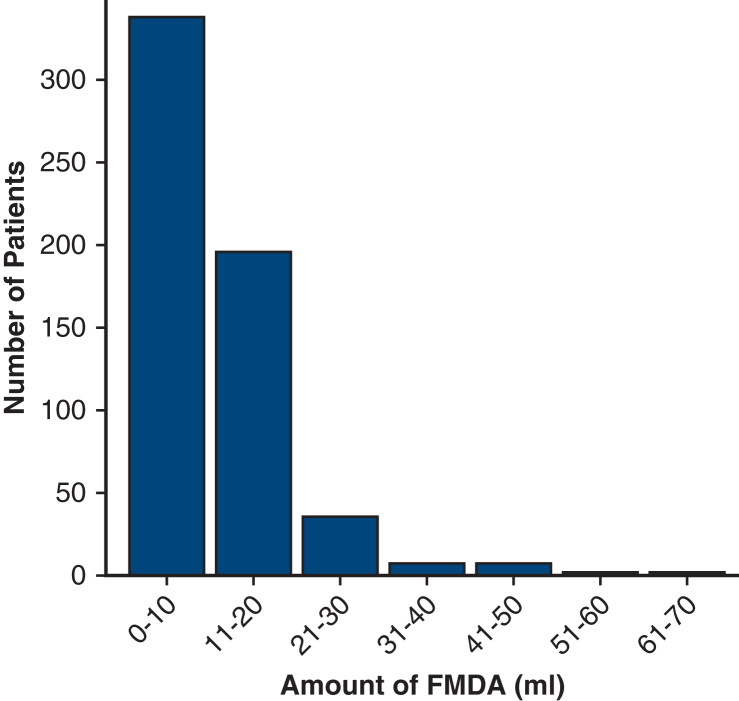
Histogram showing the distribution of the final five-minute drainage assessment (*FMDA*) volume among patients.

The perioperative variables are presented in Table 2. The FMDA group had a significantly higher rate of redo surgery compared to the control group (7.2% vs 3.5%; *P* = .003). The FMDA group had a higher proportion of MICS via right mini-thoracotomy (9.9% vs 6.6%; *P* = .040). The operative, cardiopulmonary bypass, and cross-clamp times were comparable in the 2 groups. The FMDA group had a significantly higher volume of intraoperative bleeding (mean, 1542 ± 1520 mL vs 1303 ± 1122 mL; *P* = .001) and required more transfusions of fresh frozen plasma (mean, 5.0 ± 5.8 units vs 4.2 ± 5.6 units; *P* = .009) and platelet concentrate (mean, 7.0 ± 10.0 units vs 5.0 ± 8.6 units; *P* < .001) compared to the control group. Furthermore, the FMDA group had a higher rate of intraoperative reassessment and additional hemostasis (3.2% vs 0.6%; *P* < .001). The majority of patients had a final FMDA volume of 0 to 10 mL, with progressively fewer patients in the higher-volume categories (Figure E1). The median final FMDA measurement used to determine the end of surgery was 10 mL (IQR, 5-15 mL). Among the 585 patients in the FMDA group, 534 (91.3%) met the criteria for terminating surgery, with a final FMDA volume of ≤20 mL. Among the 534 patients who met the criteria, only 5 required reexploration for bleeding, significantly fewer than the 8 of 51 patients who did not meet the criteria (*P* < .001).

**Table 2:** Perioperative variables

Variable	Control (N = 695)	FMDA (N = 585)	*P* value
Emergency surgery, n (%)	124 (17.8)	105 (17.9)	>.99
Redo surgery, n (%)	24 (3.5)	42 (7.2)	.003
Operative procedures, n (%)			
Coronary	183 (26.3)	161 (27.5)	.658
MIDCAB via left thoracotomy	3 (0.4)	5 (0.9)	.481
Valve	398 (57.3)	341 (58.3)	.733
MICS via right mini-thoracotomy	46 (6.6)	58 (9.9)	.040
Aorta	208 (29.9)	186 (31.8)	.504
Left thoracotomy	9 (1.3)	15 (2.6)	.102
Other	39 (5.6)	47 (8.0)	.093
Operative time, min, mean ± SD	363 ± 116	369 ± 121	.387
CPB time, min, mean ± SD	164 ± 103	241 ± 94.5	.110
Cross-clamp time, min, mean ± SD	101 ± 72.4	100 ± 70.8	.675
Intraoperative bleeding, mL, mean ± SD	1303 ± 1122	1542 ± 1520	.001
Blood transfusion, mean ± SD			
Red blood cells, units	3.5 ± 4.0	3.9 ± 4.6	.086
Fresh frozen plasma, units	4.2 ± 5.6	5.0 ± 5.8	.009
Platelet concentrate, units	5.0 ± 8.6	7.0 ± 10.0	<.001
Intraoperative reassessment and additional hemostasis, n (%)	4 (0.6)	19 (3.2)	<.001

*FMDA*, Five-minute drainage assessment; *MIDCAB*, minimally invasive direct coronary artery bypass grafting; *MICS*, minimally invasive cardiac surgery; *CPB*, cardiopulmonary bypass.

### Postoperative Outcomes and Complications

**Figure 3:**
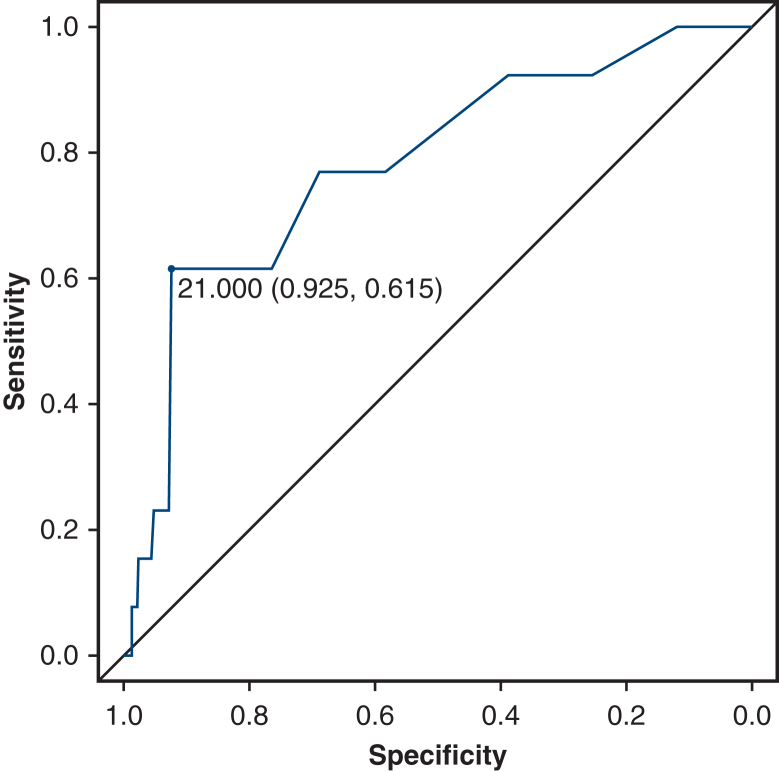
Receiver operating characteristic (*ROC*) curve for a diagnostic test showing the trade-off between sensitivity and specificity. The five-minute drainage assessment (*FMDA*) had good discriminatory ability for identifying patients at risk of reexploration for bleeding within 48 hours after surgery, with an area under the ROC curve of 0.782 (95% confidence interval, 0.645-0.918). The optimal cutoff value for the FMDA was 21.0 mL, yielding a sensitivity of 61.5% and specificity of 92.5%.

**Figure 4:**
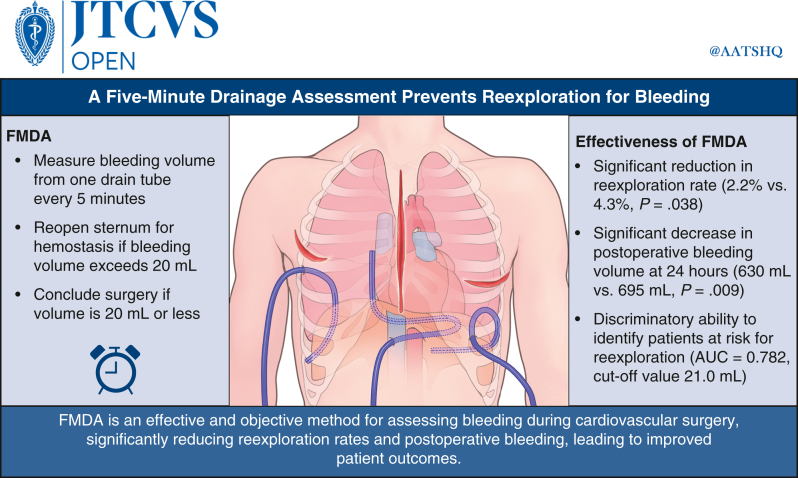
Graphical abstract of the study. *FMDA*, Five-minute drainage assessment; *AUC*, area under the curve.

**Figure E2:**
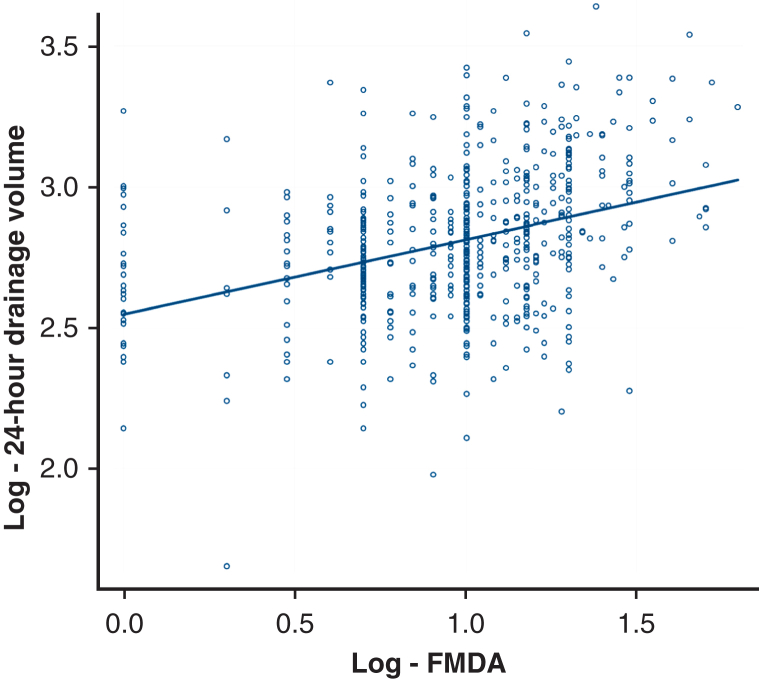
Scatterplot showing the correlation between the logarithm of the final five-minute drainage assessment (*FMDA*) volume (*x*-axis) and the logarithm of the 24-hour drainage volume (*y*-axis). The plot suggests a positive linear correlation between the 2 variables.

Table 3 summarizes the postoperative variables. The FMDA group had a significantly lower rate of reexploration for bleeding compared to the control group (2.2% vs 4.3%; *P* = .038). The rates of prolonged ventilation, deep sternal wound infection, acute kidney injury, and postoperative antiplatelet/anticoagulant therapy were similar in the 2 groups. Median 24-hour postoperative bleeding was lower in the FMDA group than in the control group (630 mL [IQR, 440-915 mL] vs 695 mL [IQR, 459-1029 mL]; *P* = .009). In-hospital mortality (1.9% vs 4.3%; *P* = .014) and suture line bleeding (1.0% vs 3.0%; *P* = .018) were less frequent in the FMDA group than in the control group. The main bleeding sites were the left atrial appendage and aortic anastomosis. Nonsutured line bleeding was similar in the 2 groups (*P* > .99) (Table E1, Table E2, Table E3). Log 24-hour drainage correlated with log FMDA (*r* = 0.363; *P* < .001) (Figure E2). Univariable analysis are shown in Table E4. Multivariable analysis showed that the FMDA independently reduced reexploration risk (odds ratio, 0.49; 95% confidence interval [CI], 0.25-0.96; *P* = .037) (Table 4). ROC analysis (AUC, 0.782; 95% CI, 0.645-0.918) indicated good discriminatory ability of the FMDA, with an optimal cutoff of 21.0 mL (sensitivity, 61.5%; specificity, 92.5%) (Figure 3). Figure 4 presents a graphical abstract of the study summarizing the main findings.

**Table 3:** Postoperative variables

Variable	Control (N = 695)	FMDA (N = 585)	*P* value
Reexploration for bleeding, n (%)	30 (4.3)	13 (2.2)	.038
Prolonged ventilation >24 h, n (%)	99 (14.2)	98 (16.8)	.243
Mediastinitis/deep sternal wound infection, n (%)	17 (2.4)	8 (1.4)	.223
Acute kidney injury, n (%)	156 (22.4)	110 (18.8)	.438
Postoperative antiplatelet therapy, n (%)	514 (74.0)	444 (75.9)	.339
Postoperative anticoagulant therapy, n (%)	376 (54.1)	300 (51.3)	.733
Postoperative bleeding within 24 h postsurgery, mL, median (IQR)	695 (459-1029)	630 (440-915)	.009
In-hospital mortality, n (%)	30 (4.3)	11 (1.9)	.014

*FMDA*, Five-minute drainage assessment; *IQR*, interquartile range.

**Table 4:** Multivariable logistic regression analysis for risk factors associated with reexploration

Risk factor	OR (95% CI)	*P* value
Age (y)	1.03 (1.00-1.06)	.053
Male sex	0.89 (0.47-1.70)	.734
Emergency surgery	1.89 (0.88-4.06)	.102
CKD (eGFR <45)	1.75 (0.85-3.59)	.128
Preoperative warfarin therapy	1.45 (0.57-3.64)	.435
Preoperative DOAC therapy	1.81 (0.74-4.40)	.191
Preoperative antiplatelet therapy	0.83 (0.42-1.64)	.593
FMDA	0.49 (0.25-0.96)	.037

*OR*, Odds ratio; *CI*, confidence interval; *CKD*, chronic kidney disease; *eGFR*, estimated glomerular filtration rate; *DOAC*, direct oral anticoagulant; *FMDA*, five-minute drainage assessment.

## Discussion

Our investigation revealed that (1) the FMDA was associated with a significantly lower rate of reexploration for bleeding following cardiovascular surgery compared to the control group; (2) the FMDA group had a significantly lower postoperative bleeding volume within 24 hours after surgery and a lower in-hospital mortality rate; and (3) the FMDA effectively identified patients at risk of reexploration within 48 hours postsurgery, with an optimal cutoff of 21.0 mL.

This study demonstrates that implementing the FMDA during cardiovascular surgery significantly reduced the rate of reexploration for bleeding and the amount of postoperative bleeding. This finding is consistent with previous studies reporting reexploration rates ranging from 2% to 6% following cardiovascular surgery.^1^^,^^2^^,^^16^ Returning to the operating room for reexploration after admission to the intensive care unit contributes to adverse outcomes,^1^, ^2^, ^3^^,^^5^^,^^6^^,^^17^ increases costs,^4^ and imposes a greater physical and mental burden on surgeons.^18^ In some cases, less experienced surgeons rather than skilled lead surgeons perform hemostasis and chest closure and make the decision to conclude the surgery. Although such experiences are necessary for surgical training, there is a need for objective and simple criteria to determine when hemostasis is achieved and surgery can be concluded. Therefore, we introduced the FMDA, which involves determining the amount of bleeding from a single drainage tube over a 5-minute period.

Comparing the postoperative reexploration rates between the FMDA and control groups (2.2% vs 4.3%) and the rate of intraoperative reassessment and additional hemostasis (3.2% vs 0.6%), both groups showed a reexploration risk of approximately 5% (5.4% vs 4.9%). However, the implementation of the FMDA may have helped avoid reexploration in approximately 3% of cases. This suggests that the FMDA may help identify and address bleeding issues earlier, potentially saving time and resources by avoiding the need to return to the operating room later.

The criterion of 20 mL in 5 minutes was based on our institution’s reopening criteria, which were determined based on several studies.^12^, ^13^, ^14^ The setting of 20 mL per 5 minutes is equivalent to 240 mL per hour, which corresponds to one of our hospital’s criteria for reexploration: continuous bleeding exceeding 200 mL per hour. This value was chosen as the threshold for the 20 mL per 5-minute setting. The ROC curve analysis revealed that the FMDA had good discriminatory ability for identifying patients at risk of reexploration for bleeding, with an AUC of 0.782. The optimal cutoff value for the FMDA was 21.0 mL. These findings suggest that the FMDA can effectively stratify patients based on their risk of postoperative bleeding, allowing targeted interventions and closer monitoring of high-risk individuals.

The higher rates of intraoperative blood loss and blood product transfusion observed in the FMDA group may be attributed to several factors. First, this group had a significantly higher proportion of patients on dialysis^19^ and higher rates of redo surgery,^20^ both of which are associated with increased bleeding risk. Additionally, implementation of the FMDA may have led to more meticulous attention to bleeding during closure, resulting in more aggressive management of even minor bleeding, which could explain the increased use of blood products in this group. In the FMDA group (n = 585), 91.3% of the patients achieved surgical closure criteria with a final volume ≤20 mL. Of 51 cases closed with ≥21 mL, 70.6% had only a slight excess (21-30 mL). This might have contributed to the overall prevention of reexploration. However, there were instances of reexploration even when the FMDA volume did not exceed 20 mL. The most common bleeding site was the left atrial appendage, and in median sternotomy cases, bleeding from the lungs was also observed, suggesting that these areas might have been difficult to aspirate owing to the positioning of the pericardial drain (Figure 2). In cases of oozing-type bleeding, the blood might not have accumulated to >20 mL during the 20 to 30 minutes of FMDA monitoring, or it might have formed a hematoma that could not be aspirated while waiting for the drainage to progress. Kunioka and colleagues^10^ reported a gauze packing method with a 1.5% reexploration rate, but it is unsuitable for MICS or thoracoabdominal surgeries and may prolong the operation time. Additionally, waiting for bleeding estimation with complete cessation of surgical manipulations may prolong the operation time. Our method involves placing a drain and determining the output, allowing simultaneous closure of the chest and surgical wounds, thereby resolving these issues. The reduction in reexploration rates can be attributed to the FMDA’s ability to accurately identify patients with excessive bleeding, allowing for timely intervention and hemostasis before concluding the surgery.

The FMDA group had lower median postoperative bleeding within 24 hours than the control group (630 vs 695 mL; *P* = .009). This reduction is clinically relevant, as excessive bleeding correlates with adverse outcomes such as increased transfusions, prolonged ventilation, and higher mortality.^8^^,^^21^ The FMDA may improve patient outcomes and reduce healthcare costs by minimizing postoperative bleeding. In the present study, we found no significant differences in adverse events like prolonged ventilation, infection, or acute renal injury between groups. However, the in-hospital mortality rate was significantly lower in the FMDA group. It is important to cautiously interpret whether this improvement can be solely attributed to use of the FMDA. Our institution's surgical outcomes have improved continuously owing to multidisciplinary collaboration and refined techniques.^22^ Therefore, multiple closely related factors likely contribute to improvements in the in-hospital mortality rate.

Multivariable logistic regression analysis identified the FMDA as an independent predictor of reduced risk for reexploration owing to bleeding. Although our observational study indicates that the FMDA may help reduce reexploration rates, we acknowledge that the study design limits our ability to establish causality. Prospective studies are needed to confirm these findings and to account for potential confounding factors, such as increased adherence to intraoperative checklists or changes in surgeons’ approach.

Limitations of this study include its retrospective design and potential selection bias. Surgeon bias may have influenced reexploration rates. The FMDA criterion of <20 mL bleeding within 5 minutes was not strictly followed; sometimes closure was done with a slightly higher volume based on surgeon's discretion and drainage color. This protocol deviation may have affected outcomes and should be considered when interpreting results. However, surgeons typically exercise caution while deciding to perform reexploration and use meticulous hemostasis techniques, and thus the effect of this bias is likely minimal.

We did not measure the total time from the start to the end of chest closure, which could have provided valuable insight into the potential impact of the FMDA on overall operative time and efficiency. We included patients who underwent thoracoabdominal aortic procedures to demonstrate the FMDA's broad applicability; however, we acknowledge that these patients may have different bleeding risks and patterns compared to other cardiac surgical patients, which could have influenced our results. Additionally, the study was conducted at a single center, which might limit the generalizability of our findings. Despite these limitations, the relatively large sample size and use of a standardized protocol for the FMDA strengthen the validity of our results.

## Conclusions

The FMDA may be an effective and objective method for reducing reexploration rates and postoperative bleeding. However, prospective studies are needed to confirm these results and establish the FMDA as a standard practice.

## Conflict of Interest Statement

The authors reported no conflicts of interest.

The *Journal* policy requires editors and reviewers to disclose conflicts of interest and to decline handling or reviewing manuscripts for which they may have a conflict of interest. The editors and reviewers of this article have no conflicts of interest.

## Figures and Tables

**Table E1:** Bleeding sites

Variable	Overall (N = 1280)	Control (N = 695)	FMDA (N = 585)	*P* value
Sutured line, n (%)	27 (2.1)	21 (3.0)	6 (1.0)	.018
Unknown and nonsutured line, n (%)	16 (1.3)	9 (1.3)	7 (1.2)	>.99

*FMDA*, Five-minute drainage assessment.

**Table E2:** Bleeding sites in the control group

Bleeding site	Number (%)
Sutured line (N = 21)	
Left atrial appendage	4 (19.0)
Aortic anastomosis	4 (19.0)
Coronary anastomosis	3 (14.3)
Left atrium	3 (14.3)
Carotid anastomosis	2 (9.5)
Right atrium	2 (9.5)
Internal mammary artery	2 (9.5)
Pulmonary artery anastomosis	1 (4.8)
Nonsutured line (N = 9)	
Wire in sternum	1 (11.1)
Arteries on the dissection surface	1 (11.1)
Intercostal thoracotomy site	1 (11.1)
Unknown	6 (66.7)

**Table E3:** Bleeding sites in the FMDA group

Bleeding site	Number (%)
Sutured line (N = 6)	
Left atrial appendage	3 (19.0)
Aortic anastomosis	2 (19.0)
Coronary anastomosis	1 (14.3)
Nonsutured line (N = 7)	
Wire in sternum	3 (42.9)
Saphenous vein graft	1 (14.3)
Arteries on the dissection surface	1 (14.3)
Lung, n (%)	1 (14.3)
Unknown, n (%)	1 (14.3)

**Table E4:** Univariable analysis for risk factors associated with reexploration

Variable	OR (95% CI)	*P* value
Age (y)	1.04 (1.01-1.07)	.016
Atrial fibrillation	2.26 (1.01-4.72)	.037
Male sex	0.72 (0.37-1.41)	.334
Hypertension	1.04 (0.50-2.41)	>.99
Hyperlipidemia	0.94 (0.48-1.82)	.878
Smoking	0.63 (0.32-1.23)	.164
Diabetes	1.24 (0.57-2.52)	.585
CKD (eGFR <45)	1.74 (0.82-3.49)	.121
Dialysis	2.05 (0.51-5.96)	.157
Previous stroke	1.03 (0.31-2.68)	>.99
PAD	1.32 (0.34-3.79)	.547
Pacemaker	1.84 (0.21-7.63)	.318
COPD	0 (0.00-2.07)	.255
Redo surgery	0 (0.00-1.64)	.165
Emergent surgery	1.61 (0.72-3.33)	.221
AAD	1.73 (0.64-4.05)	.198
MICS via right thoracotomy	0.26 (0.01-1.58)	.251
MIDCAB via left thoracotomy	0 (0.00-17.3)	>.99
Aorta-left thoracotomy	0 (0.00-4.88)	>.99
Coronary	1.33 (0.64-2.63)	.390
Valve	0.84 (0.43-1.63)	.638
Aorta	1.09 (0.53-2.16)	.867
Other	1.44 (0.37-4.15)	.527
Pre-warfarin	1.52 (0.51-3.74)	.302
Pre-DOAC	1.72 (0.63-4.01)	.201
Pre-antiplatelet therapy	0.89 (0.43-1.75)	.871
FMDA	0.50 (0.24-0.99)	.043

*OR*, Odds ratio; *CI*, confidence interval; *CKD*, chronic kidney disease; *eGFR*, estimated glomerular filtration rate; *PAD*, peripheral artery disease; *COPD*, chronic obstructive pulmonary disease; *AAD*, acute aortic dissection; *MICS*, minimally invasive cardiac surgery; *MIDCAB*, minimally invasive direct coronary artery bypass; *DOAC*, direct oral anticoagulant; *FMDA*, five-minute drainage assessment.

## Supplementary Data

Video 1 Demonstration of the five-minute drainage assessment (FMDA) procedure during cardiovascular surgery. The video shows placement of the drainage tube in cases of median sternotomy, collection of blood over a 5-minute period, and the decision making process based on the collected volume to determine whether to end the surgery or perform additional hemostasis. Video available at: https://www.jtcvs.org/article/S2666-2736(24)00230-4/fulltext.
