# Supplementary figures and images for: A five-minute drainage assessment prevents reexploration for bleeding
Source: JTCVS Open. 2024 Aug 27;22:65–75. doi: 10.1016/j.xjon.2024.08.008 (PMC11704565; doi:10.1016/j.xjon.2024.08.008)

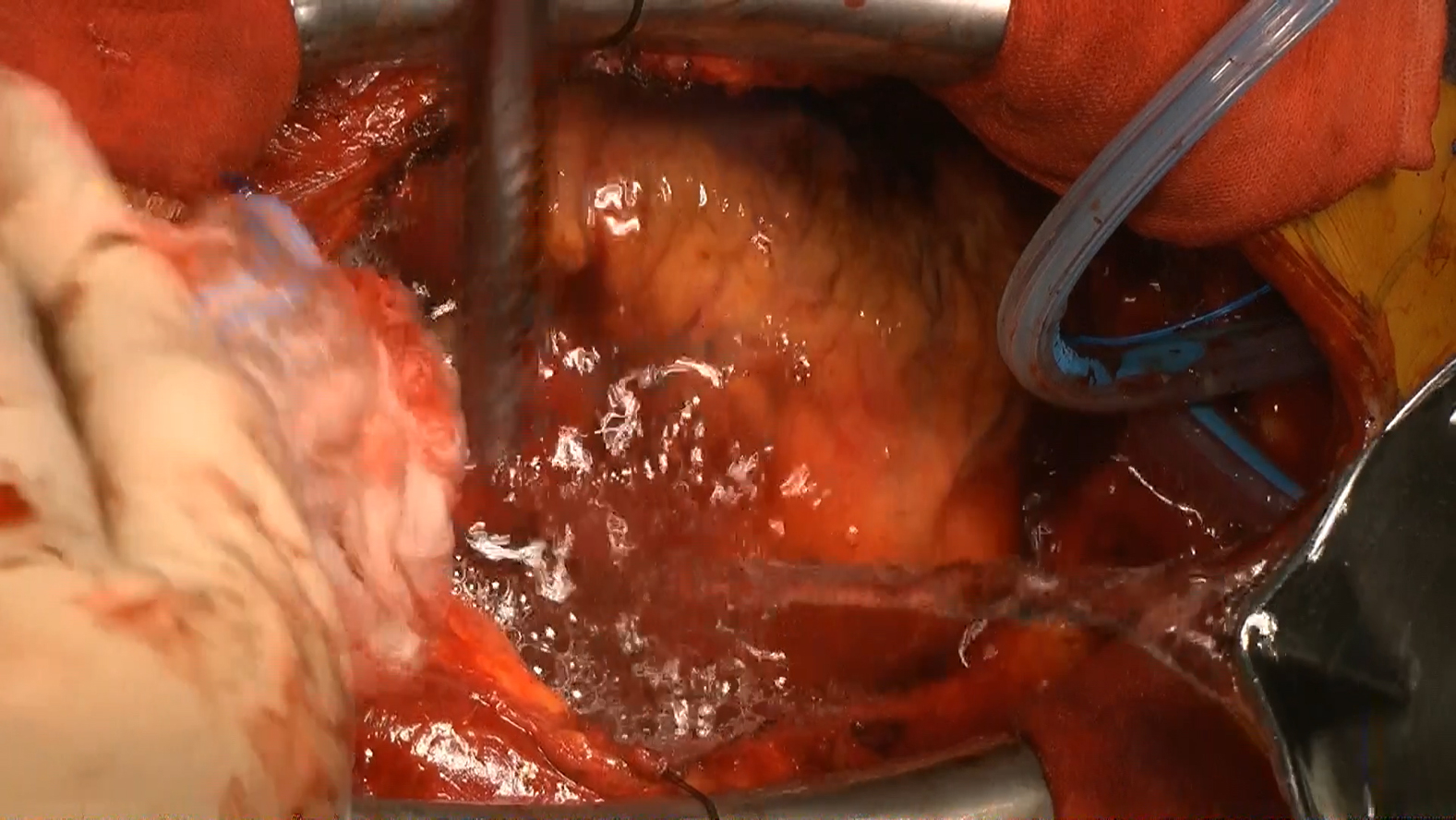

Supplement: Video 1 — Demonstration of the five-minute drainage assessment (FMDA) procedure during cardiovascular surgery. The video shows placement of the drainage tube in cases of median sternotomy, collection of blood over a 5-minute period, and the decision making process based on the collected volume to determine whether to end the surgery or perform additional hemostasis. Video available at: https://www.jtcvs.org/article/S2666-2736(24)00230-4/fulltext. [file fx2.jpg]
